# Lectures on the Diseases of Women

**Published:** 1859-03

**Authors:** 


ART. II.—*Lectures on the Diseases of Women.* By CHARLES WEST, M.D., Physician Accoucheur to St. Bartholomew's Hospital, and Physician to the Hospital for Sick Children. Part II.: Diseases of the Ovaries, Vagina, Bladder, and External Organs. 8vo., pp. 309–490. Philadelphia: Blanchard & Lea, 1858.

A REVIEW of the first part of this work was presented in our July number for last year, and we congratulate the profession upon the appearance of the second part, as it completes a valuable work upon an important branch of practice, and is the production of a man of great ability and wide experience. Our remarks there preclude the necessity of dwelling here upon Dr. West's characteristics as a writer or a teacher; he has been for some time before the profession; and in commencing our examination of the present work, we need only remark that it presents the same felicity of style, the same candor of examination, the same caution in statement, and the same absence of anything savoring of partisanship upon questions not yet decided, which characterized his former volume. The results of his own experience in each disease are given so clearly and carefully as to make his works specially valuable in this respect; he says, in the preface to the present volume, "I have not ventured to teach concerning matters with reference to which I feel myself to be still altogether a learner; while I have always regarded mere compilation, uncontrolled by experience, as more apt to perpetuate error than to diffuse truth." He shows, too, such a modest distrust of himself where his experience conflicts with that of other eminent men in the same branch of practice, that while the reader is charmed with a trait so pleasing and so rare, the conviction is forced upon him that he is following a careful and judicious guide.

The first part of the work treats of diseases of the uterus; this volume comprises affections of the other female sexual organs. The first two of the twelve lectures it contains are occupied with the inflammation and consequent abscess of the uterine appendages, and with uterine hæmatocele; the last three are upon diseases of the bladder, vagina, and external organs. In the seven remaining lectures diseases of the ovaries are discussed, and these so far exceed all others in interest and importance, that we shall occupy what space we have at our disposal in examining the views of the author upon the different points connected with them.

One lecture is taken up with the subject of inflammation of the ovaries, acute and chronic, and serves as an introduction to the more important diseases of these organs. But a single case of acute inflammation has come under the author's observation, and he does not therefore occupy much space with this disease, but in regard to the chronic form, the most important fact is that he cannot coincide with those who look upon it as a serious disease, occasioning much local and constitutional suffering, giving rise to great disturbances of menstruation, and being the most frequent cause of sterility. Pain, he says, is not so correct an index of the extent and nature of diseases connected with the sexual diseases of women, as in other cases; nor does the appearance of the tissues after death warrant the belief that chronic ovaritis is a frequent disease, or one causing so many different ailments during life. He can no more agree with those who ascribe so many female complaints to chronic ovaritis, than with those who attribute them to ulceration of the cervix uteri; he agrees rather with Churchill in calling a certain class of cases in which the principal symptom is pain in one or the other iliac region, aggravated during menstruation, as depending upon ovarian irritation, although he prefers the simpler term of *ovarian pain.*

The lectures upon ovarian tumors and dropsy are the ones to which every reader will turn with the greatest interest. To this subject as much attention is now paid as to any other in the domain of medicine,—the difficulties inherent in the subject from the differences in the form of disease, the different conclusions to which different and seemingly equally qualified observers have arrived, and the warm partisanship felt by many advocates of the different remedial measures proposed, have all conspired to make everything upon the subject coming from a good source of the greatest interest to the profession. This importance and these difficulties of the subject which we have glanced at, and which rush into the mind of every professional reader the moment he turns his attention to the subject, are so well presented in the introductory remarks that we cannot avoid quoting them :—

"They occur in the young and the aged, in the single and in the married, in the sterile and in women who have given birth to many children. They are formed, sometimes, by simple cysts containing serous fluid; at other times they are composed of solid matter; while in very many instances their structure is identical with that of growths which morbid anatomists have unanimously designated malignant. Their rate of increase is sometimes quick, at other times slow, and the disease which had seemed in course of rapid development becomes occasionally stationary, and so remains for months or years; while now and then nature herself interferes, and excelling all that the most skillful physician could do, completely takes away the ill which medicine is usually impotent to cure. Their diagnosis, in some cases most easy, is in others attended by extreme difficulty; and yet there are scarcely any ailments in which so much is involved in a right decision. The determination that the supposed disease is in reality due to the existence of pregnancy, or that the suspected pregnancy is but the evidence of disease, often has moral consequences which touch more nearly the profoundest source of human happiness or misery than any which would follow the mere assurance, though never so positive, of coming health, or the admission that the future has no other prospect than that of a lingering and painful death. The prognosis to be formed, and the treatment to be adopted, bring with them, too, their own peculiar difficulties. Recovery, when there seemed no ground for hope; death, when little had appeared to call for apprehension; medical treatment rejected because it had been proved inefficacious; surgical proceedings shrunk from, because they are known to be hazardous; additional facts scarcely seeming to widen our experience, or serving only to detect the fallacy of some loudly-vaunted plan of cures,—such are the uncertainties, and such the difficulties that meet us when we propose to ourselves the inquiry—What shall we do? In short, there are no diseases whose pathology is more imperfect, whose symptoms are more fluctuating, whose diagnosis is more obscure, or whose treatment is founded on more uncertain data, than those very diseases of the ovaries, which are yet so important, and to whose study I must now beg to call your most patient attention."

Leaving this general sketch of the subject—as faithful as it is sad—let us turn to particulars. Some of these we shall only glance at; with many we shall not occupy any time at all. Upon the course and causes of the disease, its influence upon sexual health, the manner in which they prove fatal, and the different modes in which nature, though rarely effects a cure, the work contains full details, but they need not detain us here. In classification the author follows Paget, and treats of these tumors under the two divisions of Simple, or Barren Cysts, and Compound, or Proliferous Cysts; this plan he thinks not only "anatomically correct, but practically convenient," and he describes at length the different forms of the disease from their origin through all the stages of their development; a description which corresponds with the twenty-second and twenty-third lectures of *Paget's Surgical Pathology.*

In regard to the relative frequency with which disease attacks either ovary, a table is given, which may be found on page 850 of the first volume of this journal. This table exhibits a very great preponderance of disease in the right ovary, but is stated to show only a very rough approximation to the truth on this point, as the sources of fallacy are so great during the life of the patient, that no conclusion can be relied upon which is not based on post-mortem examination. The result is therefore given of forty-one cases by Scanzoni, fifteen by Dr. R. Lee, and nineteen by the author, in which the condition of the parts was truly ascertained by examination after death; these seventy-five cases show twenty-six of disease of the right side alone, twenty-three of the left, and twenty-six in which both ovaries were affected; the author does not therefore believe that there is any special liability of one ovary to disease more than the other.

Scanzoni is stated to be the only author who has attempted an estimation of the relative frequency with which the different forms of ovarian disease appear; to his forty-one cases Dr. West adds nineteen of his own, and the result is shown in the following table:—

| | |
|---|---|
| Simple cysts . . . . . . . . . . | in 15 cases. |
| Fat cyst . . . . . . . . . . . | in 1 case. |
| Compound cysts and cysto-sarcoma . . . . | in 23 cases. |
| Colloid, or alveolar tumors . . . . . . | in 19 cases. |
| Cancer with cyst formation . . . . . . | in 2 cases. |
| Total . . . . . . . . . . . . | 60 cases. |

The symptoms of ovarian tumors and dropsy are considered at length under five heads: functional disorder of the organs as shown by various derangements of menstruation, pain, the effect of pressure, and the cachexia, which is so striking an accompaniment of some forms of disease; the fifth division, including those symptoms which arise from our efforts to cure the disease, or to prolong the life of the patient, such as peritonitis and cyst-inflammation following tapping. In treating of diagnosis, the points of difference between these diseases and others likely to be confounded are fully and plainly stated; particularly in regard to inflammation of the broad ligaments and its consecutive abscesses, fibrous tumors of the uterus, misplacements of that organ, distension of the bladder, and tumors of the liver and spleen, while rarer forms of disease which have sometimes occasioned mistake are mentioned, and the means of distinguishing these tumors from the physiological condition of pregnancy is not of course forgotten. There is probably no disease coming under our professional care in which diagnosis is more difficult than in the one under consideration; we have not merely to distinguish the tumor from diseases of other organs, and can neither satisfy ourself nor our patient by the statement that it is the ovary which is affected, but must make such a diagnosis as will in some degree serve as a foundation for prognosis, and if we are further to undertake any surgical proceedings for its cure, the highest considerations which can affect ourselves and our patient demand that there shall be no error in regard to the nature of the disease. Upon this point the author is earnest and impressive, warning us not to take any information second-hand, but to rely alone upon that obtained ourselves, and by careful and repeated examinations:—

"That fæces in the large intestines have been taken for them; that fat and flatus have raised a suspicion of their presence; that the abdomen even has been opened to remove a tumor which was found to have no existence, proves only how large is the possibility of error, how vigilant must be our care, if we will avoid a danger which the wisest have not always been so fortunate as to escape."

The fact is stated, among other instances of mistake, that Boinet once injected a solution of iodine into the peritoneal cavity with fatal result, supposing he was dealing with a case of ovarian dropsy; but far more striking, and far more likely to be remembered, is a "painful case" which occurred under the author's observation, the details of which are given. The patient was a girl about seventeen years of age, who was admitted into St. Bartholomew's Hospital, supposed to be suffering from ovarian dropsy, with which diagnosis the signs, negative as well as positive, seemed to agree perfectly; at the second tapping ten ounces of a solution of iodine were injected, which produced fainting and other alarming symptoms, and caused death in sixteen hours. The post-mortem examination showed cirrhosis of the liver, which had produced ascites, while the uterus and its appendages were healthy, and no tumor was to be found. The occurrence af such a case in a metropolitan hospital, with a corps of experienced surgeons and obstetricians, most forcibly teaches, as the author well remarks, the sleepless watchfulness necessary to guard from error, and "the importance of not taking anything upon trust, nor of allowing our judgment to be swayed by any previously expressed opinion as to the nature of the disease."

The treatment of the disease is opened cautiously, and a deep feeling is shown of the difficulties which surround it, arising from the nature of the complaint itself and from the conflicting experience of different observers. In continuing the subject he exhibits such a manifest desire to state fairly the merits of each plan, such an absence of all partisanship, and makes such candid admissions of the necessity of more facts to enable us to judge definitely of many points, that the warmest advocate of any particular plan of treatment cannot find fault, while the opinions expressed are so well sustained by statistics that the unbiased reader will not fail to be convinced of their truth. He lays down the principle in the beginning, that the danger of a disease should never be lost sight of in judging of the expediency of interference,—that if present suffering is not great, and a rapid advance not certain, "we should hesitate to advise any proceeding by which, though perfect cure may possibly be wrought, yet, on the other hand, life may be cut short suddenly." The statistics of the duration of the disease are then given, and they show that in sixteen out of one hundred and seventy-three cases, nearly one in seven, life continued from ten to fifty years after the commencement of the disease; it is therefore evident that "one year and fifty years cannot both truly represent the time occupied by the same disease in running its course," and it shows what morbid anatomy had done before, "that under this name several different diseases have been included, having different tendencies, warranting a different prognosis, and calling for different modes of treatment." In viewing this fact with reference to any particular mode of treatment he continues:—

* * * "We must consider it with reference to the special form of the affection with which, in each separate case, we have to do. The question cannot be propounded as to whether this or that plan of treatment is suitable for ovarian dropsy; but, given a certain form of ovarian disease, is this or that proceeding expedient or allowable; or is it easier to do nothing, or to palliate; or is the attempt to do more justifiable; and when at length the necessity for interference of *some* kind becomes absolutely unquestionable, are the risks even of palliative proceedings so considerable as to warrant a greater hazard being run for the chances of a perfect cure?"

The classification of these cases for treatment is not based by the author upon any particular symptoms present, nor wholly upon the anatomical character of the tumor, but is entirely arbitrary and consists of three classes:—

1. Cases which *may* be let alone.
2. Cases which *must* be let alone.
3. Cases *justifying*, or *absolutely requiring interference*.

These cases belong to the first class in which the tumor does not exceed the size of the two fists, does not seriously disturb the functions of the pelvic viscera, does not cause much suffering, and is not rapidly increasing. But by *letting alone*, the author does not mean that nothing at all is to be done for such cases, and that we are to abandon the patients, trusting the issue to fortune. There are two indications to be fulfilled which are so important as to deserve to be constantly kept in mind and steadily pursued; the general health should be carefully maintained, and congestions of the pelvic viscera prevented by every possible means. Both require hygienic measures more than medicines:—

"The first indication, I conceive, implies the avoidance of all such proceedings as courses of mercury, of iodine, of iodide of potass, or of liquor potassæ, agents of whose power in retarding the development of ovarian cysts, there is scarcely any evidence, while of their injurious influence on the constitution, when long continued, there is the most abundant proof."

The second indication requires unusual care during the menstrual periods, that the physiological congestion of the pelvic organs, then taking place, may be restrained as much as possible, by attention always to the state of the bowels, leeching when requisite, and the application of a properly-fitting bandage.

The cases of ovarian tumor which *must* be let alone, are those, "for the most part, of rather rapid growth, whose irregular nodulated surface and whose solid non-fluctuating mass suggests the idea that they are not mere compound cysts, but productions of a malignant character." In these cases there are also present evidences of a marked cachexia; the general health was broken down before the tumor had attained sufficient size to interfere with nutrition, either by the amount of sustenance withdrawn from the circulation for its own growth, or by interfering with the functions of the stomach by pressure. This class is speedily dismissed with the sad reflection that "the cases which seem most to call for help are those in which it is least possible to afford it, while it is in precisely those which may most safely be let alone that interference has the best chance of success."

The consideration of the class between these two extremes, consisting of those cases which justify or demand interference, introduces the more decided measures which have been resorted to for the cure of the disease or the prolongation of the life of the patient. The different views of the profession upon the period of time at which tapping is justifiable are first given; some being influenced by the immediate dangers of the operation, the rapidity with which the cyst refills, and the very small number of cases in which it proves curative, postpone it until compelled by the urgent symptoms arising from the size of the tumor; others relying more upon the few cases of cure and the many in which, by being frequently repeated, it has prolonged life to its usual extent, resort to it at an early period. But in fact the question cannot be considered or decided upon alone; to simple tapping has been added the injection of the cysts with iodine, and other measures have been proposed and put in force to effect a radical cure. Until the relative mortality and the proportion of cures of these different modes are given, we cannot decide upon the time at which tapping may be performed with the greatest benefit to the patient. The author acknowledges that future experience may modify or completely change his opinion upon this point, and gives the following as his present views:—

"My present belief, however, is that the dangers of the operation have on the whole been over-estimated; and further, that while in cases where the amount of solid matter in the growth is considerable, the rule which prescribes the postponement of the operation to the latest possible period is a sound one; it will probably be more expedient in the case of simple ovarian cysts to tap early, before the growth had acquired a large size, and before the constitutional powers of the patient have seriously suffered."

The immediate dangers of tapping are twofold: *shock* and *inflammation of the cyst.* These are both considered at length, and tables are given showing the length of life after the operation and the relative mortality of each repetition of its performance; of 130 cases derived from other writers, 34·7 per cent. proved fatal in the course of six months after the operation; of thirty-one cases under his own care, three proved fatal very soon. But an important caution is here given in regard to deducing any conclusion from hospital statistics; the class of persons who apply to such institutions for relief; the advanced stage of disease in which most of them enter; the disappearance of the cures, while the failures are sure to be noted, all tending to increase the unfavorable side of the question,—render statistics, drawn from hospital experience, unreliable as guides to the truth of any question concerning the mortality of a given operation.

"Almost in proportion as experience concerning this operation is derived from hospital practice, or from observation in private, does the estimate of its danger appear to be increased or lessened, a circumstance which seems to show that the hazards of the operation depend at least as much on the conditions that surround the patient as anything inherent in the proceeding itself."

There are five modifications of tapping which have been proposed to effect that which the simple operation cannot be relied upon to do—a radical cure of the disease. We shall give the opinion of the author upon each of these:—

I. *The application of light bandages after the evacuation of the cyst.* This mode has been pursued by Dr. Baker Brown, who at first used active diuretics and other medication in addition to the mechanical means. As a means of retarding the re-accumulation of the fluid, Dr. West thinks well of it; but as a curative measure, can place no reliance upon it. It is requisite to persevere in it for some time, and is said to be peculiarly adapted to simple cysts—just the class of cases, as the author remarks, in which a cure is most likely to follow simple tapping, and of which his experience give two instances.

II. *The subcutaneous puncture of the tumor* has been suggested, upon the supposition that the fluid would be absorbed by the peritoneum without danger to the patient; but the fluid is often such as to render this impossible, while any exploration for the purpose of ascertaining its nature violates the conditions of the proposed mode of cure by the admission of air.

III. *Puncture of the cyst through the vagina.* The peculiar advantage of this plan is that from the dependent position of the wound in the cyst, its contents are completely evacuated and thus adhesion is far more likely to follow and a permanent cure result. It is best adapted to simple cysts of a moderate size. The objections are stated to be—1st. The danger of wounding the bladder, from its altered shape and changed position, is very great, "with no very great unskillfulness on the part of the operator". 2d. In compound cysts there is a much larger amount of solid matter at the lower part of the tumor, and we may not therefore succeed in emptying it so well as through the abdominal walls. 3d. From the arrangement of the blood-vessels the danger of hemorrhage is much increased by puncture near the pedicle.

IV. *Keeping the cyst empty* either by means of a tube or by a fistulous opening. There are three modes of effecting this—the tube being passed through the vagina, through the abdominal walls, or the fistula being made through them, and the excision of a portion of the cyst. The first was the favorite method of Kiwisch, and was followed by his successor Scanzoni, who speaks so highly of it in his *Handbuch der Krankheiten der Weiblichen Sexualorgane,* and gives such favorable statistics of it, that we ardently hoped to find corroborative testimony of its value. Scanzoni mentions fourteen cases, in eight of which a perfect cure followed this mode of treatment; three of the patients were lost sight of, one died of typhus fever two months after the operation, and death did not result from it in any case. The author has operated in this way upon three cases, one of which proved fatal from consecutive inflammation, one was lost sight of after two months, during which time there was no re-accumulation of fluid, and the other remains in good health with a fistulous opening from the cyst to the vagina. With the plan of opening the cyst through the abdominal parietes and stitching its edges to them, the author's experience extends to two cases, in both of which it was the termination of an incision made with the intention of extirpating the disease, had not adhesions prevented; both cases proved fatal, one in ninety-six hours, the other in seventeen days. For information in regard to excision of a portion of the sac, reference is made to the writings of Brown, Atlee, and others, as no cases have come under his observation.

V. *Iodine injections into the sac.* Occasional notices in the journals of cases treated in this way, at St. Bartholomew's, have naturally excited an interest to learn the author's views upon it, and the results of his experience. It is his favorite plan, his experience, and that of others, showing a less number of deaths, and a greater proportion of cures, than by any other mode of treatment, although it is admitted that further experience and more precise information are necessary to settle accurately its relative value, which is not surprising, since ten years have not yet elapsed from the publication of the first reports of cases. The statistics of Boinet are given in full; they show the result of forty-five operations on forty-four patients, of whom twenty-eight were between the ages of thirty and fifty, nine below the former age, and eight above the latter. These cases gave thirty-one cures, five failures, and nine deaths.

"In thirty-four of the cases the cysts were simple; in eleven, compound. All the successes occurred where the cyst was simple; but three deaths also followed the injection of simple cysts. All the operations on compound cysts failed; and six of them were followed by the patient's death; though certainly in many of these cases death would have taken place as soon, possibly even sooner, if interference had not been resorted to."

The author has seen eight cases of iodine injection, the full particulars of which are presented in a table. A positive cure is reported in but one case, while in two others no re-accumulation took place until after eighteen months or two years; the operation did not cause death in any case under the author's observation.

The injection recommended for use consists of five parts of iodine, five of iodide of potassium, fifty of spirit, and one hundred of water, prepared at the time it is to be used. The presence of spirit is supposed to retard the absorption of the active agent, and, therefore, to prevent or lessen the consequent *iodism*, characterized by great abdominal pain, cold extremities, a frequent and feeble pulse, and all the symptoms of extreme depression. As another means of avoiding these dangerous symptoms, he removes the injection after it has remained ten or fifteen minutes, being decidedly opposed to the plan pursued by some, of leaving it in the cyst.

The very favorable results obtained by Simpson and Velpeau with this mode of treatment are alluded to, and regret expressed that the former has not stated his experience definitely enough to be available in a numerical statement. The objections of Scanzoni are considered; and in summing up the author acknowledges himself much pleased with iodine injections, and anxiously awaiting that which he acknowledges to be necessary—further experience, and especially an increase of a knowledge in regard to the discrimination of cases to which they are best adapted.

"The uncertainty as to the cases which will bear the iodine injection well, as distinguished from those in which cyst-inflammation or profound iodism will be excited by it, is a drawback from its value which this operation shares with every other proceeding for the cure, or even for the temporary relief, of ovarian dropsy."

In commencing the subject of ovariotomy, the last great remedy for the radical cure of the disease, a sketch of the history of the operation is given, and the author then plunges into statistics. He gives credit to Dr. Atlee, of this country, for having published the first table of cases upon this subject, and for having furnished much valuable information; Fock, a German writer, is, however, his great authority, having published a collection of all reported cases up to eighteen months before the time of writing. This collection gives 292 cases, in 200 of which extirpation was performed, and in 92 only attempted. The following is the summing up:—

* * * "These 200 cases of actual extirpation of the ovary yield 111 recoveries to 89 deaths; or, in other words, the mortality is $44\frac{1}{2}$ per cent., or not very far short of half the number of persons in whom the operation is completed die from its effects. But, besides these, there are 92 cases in which the operation could not be completed, on account of the presence of adhesions, or of the tumor having some other situation or other attachments than was supposed beforehand, or in which some even greater diagnostic error was committed, and the very existence of the tumor was found to be a mistake. Of these 92 patients 31 died, or 33·6 per cent., or 1 in every 3; but 9 of those who survived, after passing through great perils, are reported to have been more or less completely cured of the disease. Putting all the cases together, it seems that of 292 recorded instances of the operation being attempted, 120 ended in death, and 92 in failure; or, in other words, the chances are two to one that the operation will be accomplished; but, if it succeeds, they are nearly equal that the patient will die; and if it fails, the prospect of her surviving the fruitless interference is only double that of her sinking in consequence of it."

A few pages farther on, where the author compares the mutability of this operation with that of the Cæsarean section, a note is appended, containing the results of all the cases of ovariotomy which have been performed in Germany, a country where, for various reasons, it is "probable that all cases, unfavorable as well as favorable, will be reported in greater proportion than elsewhere." The cases are 64 in number, and they yield 12 radical cures, 46 with fatal issue, and 6, the "benefits of which were either questionable, temporary, or turned out utter failures;" thus giving a mortality of 72 per cent. compared with 63; or, according to some, 66·6 by the Cæsarean operation. Nor has the experience of the author been any more favorable; he has seen five cases, two of which are here published for the first time; in all these cases "the operation was undertaken after much consideration, with the approval and under the direction of surgeons of large experience and undoubted skill, but who in every instance were baffled in their attempt."

In continuing the statistics, tables are given of the time at which death occurred after the operation, the different accidents which caused it, the ages of the patients, and of other particulars of interest. The opinion held by some advocates of the operation, that the mortality is in course of diminution, and that the future will show more cures than the past, is then examined; from the figures shown by later records, compared with the earlier ones, the author cannot think this view correct. It is reasonable to suppose that such improvement will be more personal than general—the individual operator who is favored by the number of cases which may fall into his hands, may be able to point with pride to convincing evidences of increased skill, but the nature of the operation seems to preclude that diminished mortality from its performance, which is the case with other operations, dexterity in which can only be acquired in the dissecting-room.

The relative mortality of the two modes of operation, by the large and by the small incision, is next considered, the statistics of Lee, Atlee, and others, being given. It has been held by the advocates of the latter that the mortality is much less than by the former; to this view the author also dissents, and thinks that while early statistics gave very favorable results, later ones show little, if any, difference between the two. It is the amount of interference which has far more influence in deciding the fate of the patient than a few inches more or less of external incision; this is strikingly shown by tables of the comparative results in simple and compound cases, of which we append one:—

| | Recoveries. | Deaths. |
|---|---|---|
| Simple cysts . . . . . . . . . . | 31 | 12 |
| Compound cysts, cysts with solid matter, and solid tumors . | 62 | 56 |
| | 93 | 68 |

The absolute and relative mortality of the operation having been given, and every fact presented which can be expressed by figures, a conclusion is drawn unfavorable to the operation of ovariotomy, upon three grounds:

1. The rate of mortality not diminishing; and,
2. The high rate of that mortality;
3. The utter impossibility of forming a prognosis in any given case.

"To the best of my knowledge, there is no other operation in surgery concerning which the chances are nearly one in three that some unforeseen difficulty will prevent its completion, or that a third of the abortive attempts at its performance will end in the patient's death."

The arguments advanced by the advocates of ovariotomy, that the rate of mortality from it is no greater than from some other operations which are frequently performed, is then considered, and we think dealt with most judiciously. The ground is taken that there can be no comparison between operations essentially dissimilar in their nature and in the circumstances under which they are performed; the mortality from ovariotomy can, therefore, no more be compared with that from amputation at the hip-joint or ligature of the subclavian artery, than with that resulting from tracheotomy in croup. Without pressing the strong points that most other operations can be completed while this must often be left unfinished, that the dangers of other operations can be estimated beforehand, while the greatest difficulties in this are often not discovered until it has been commenced, he thinks that ovariotomy should be tested rather by a comparison of its results with tapping, iodine injections, and other modes of radical cure, rather than with those operations which resemble it only in the high rate of mortality attending their performance.

We have said that the decision of the author is adverse to the operation of extirpation of the ovary. Should it be concluded, therefore, that he is opposed to its performance under any circumstances, and expressed his decision with the tone of one who felt that there was nothing which had eluded his research or escaped his observation, the utmost injustice would be done him. The candor with which he acknowledges that further experience and more careful study are necessary before the question can be settled, is an excellent example of one of his best characteristics. We have already seen that his opinion is formed in great part upon the statistics of the disease, and he evidently feels that while figures are admirable guides to a general result, they are of little value at the bedside in forming the prognosis of the particular case then before us. "*Non numerandæ, sed perpendendæ sunt observationes,*" said Morgagni, long ago, and the saying is especially applicable to ovarian disease. The adverse decision is, therefore, not an absolute one, nor one given as upon sufficient grounds to serve as the expression of the profession in general, but as the conclusion of the individual upon the facts at present collected, while he admits that further experience and observation are absolutely necessary. Until these are obtained the operation must take its place "by the side of those exceptional proceedings, the expediency of which must be determined by each one for himself after a careful consideration of the peculiarities of the case and the idiosyncrasies of the patient." In this respect he presents a marked contrast to Scanzoni, whose denunciation of the operation and those who undertake it may be found on p. 855 of the first volume of the Review; the desire of the patient for the operation, after the facts were candidly presented to her, would not, with him, have any weight, or be any justification for an attempt at extirpation; yet, to our mind this cannot be thrown out of the scale, so long as we allow to patients the absolute right to refuse an operation, such as amputation of an injured limb, where we can assure them almost positively that the operation will save life. But for the benefit of all who are inclined to ultra views upon this or any other subject, we quote the concluding paragraph of the lectures on ovariotomy:—

"One remark I cannot refrain from making in conclusion on the grievous injury that is done both to the advance of medical knowledge, and to the standing of our profession with the public, by the practice of treating some of these questions as though they were questions of moral right or wrong. It would seem, from what has sometimes been said on the subject, almost as if ovariotomy could not be defended save from some sinister end, nor its expediency be doubted except from a moral obliquity rendered excusable only by hopeless dullness. Belief in each other's integrity of purpose seems to me essential to our eliciting truth by discussion; and I see no reason why I am to suspect another of being less mindful of our commom duty to humanity, because he tries to relieve suffering or to prolong life by some means in which I have not the same confidence. The *odium theologicum* has at least age and respectability in its favor; I fear the immortal quarrel between Dr. Slop and Susannah has gone far to render the *odium obstetricantium* simply ridiculous."

So near the end of our task, we are sorry to be obliged to find fault: yet complete as has been our satisfaction heretofore, there is one point which justice to individual and national reputation compels us to mention. We turned to the subject of vesico-vaginal fistula with the most lively interest, as we suppose nine-tenths of the readers in this country will do, and were deeply disappointed to find its treatment dismissed with a single paragraph, and without any mention of what is justly considered one of the greatest improvements lately made in surgery. That details of the surgical proceedings necessary for the cure of this disease were omitted did not surprise us, for they are not within the author's plan, but a course of lectures, printed for the benefit of students as well as practitioners, should certainly contain some notice of all considerable advances lately made in the branch of which they treat, and indicate where such information may be found, as it is not in their province to furnish. That Dr. Bozeman's button-suture is not mentioned, nor any indication given that any advance has been made in this operation beyond the improvements made by Dr. Sims, from whom we have not the slightest wish to withhold the honor which he so justly deserves, is cause for great regret, and we believe will be deeply felt in this country. The omission is unaccountable, for the operation had been performed in London by Baker Brown and others, before the concluding chapters could have passed through the press, and we confess ourselves unable to frame an explanation which carries with it a reasonable amount of probability.

We trust that this is not the last time we shall be called upon to notice Dr. West's labors as an author; his talents and his opportunities are too great to be confined to the walls of a hospital or the limits of a class of students, and the facility with which he expresses his knowledge renders it doubly desirable that he should continue to work for the benefit of the profession at large. We consider his volumes on Diseases of Women a most excellent treatise upon that branch of practice, and, with the one exception just mentioned, accord it our unqualified praise.

